# Supplementary material for: Biodegradation of Polystyrene by Galleria mellonella: Identification of Potential Enzymes Involved in the Degradative Pathway
Source: Int J Mol Sci. 2024 Jan 27;25(3):1576. doi: 10.3390/ijms25031576 (PMC10855133; doi:10.3390/ijms25031576)
Supplement: Supplementary file 1 [file ijms-25-01576-s001.zip › ijms-2805945-supplementary.pdf]

Biodegradation of polystyrene by *Galleria mellonella*: identification of possible enzymes involved in the degradative pathway

Sebastian Venegas[a], Carolina Alarcon[a], Juan Araya[b], Marcell Gatica[a], Violeta Morin[a], Estefania Tarifeño-Saldivia*[1a], Elena Uribe*[2a].

[1] etarisal@udec.cl
[2] auribe@udec.cl
* Co-corresponding authors
[a] Department of Biochemistry and Molecular Biology, Faculty of Biological Sciences, University of Concepción, Chile.
[b] Department of Instrumental Analysis, Faculty of Pharmacy, University of Concepción, Chile.

*Correspondence: Estefanía Tarifeño 56-41-2204428, e-mail: etarisaludec.cl, Elena Uribe, 56-41-2204428, e-mail: auribe@udec.cl.

Results Section

Table S1. Band assignment of Control treatment frass spectra.

Component 1 Band Position (cm^{-1})	Component 2 Band Position (cm^{-1})	Component 3 Band Position (cm^{-1})	Type of vibration
3279 (m,b)	3162 (w,b)	3270 (m,b)	ν(O-H) in Alcohol
		3270-3030 (m,b)	ν(O-H) in Carboxylic Acid
2922 (m)	2919 (st)		ν_{as}(C-H) in Alkane
		2887 (m)	ν_{s}(C-H) in Alkane
2850 (m)	2850 (m)	2808 (m)	ν_{s}(C-H) in Alkane
1734 (w)	1737 (m)		ν(C=O) in carbonyls
1623 (w)		1638 (st)	ν(C=O) in Amide
1533 (w)		1536 (st)	ν(N-H) and ν(C-N) in Amide
1449-1239 (w)	1464-1242(w)	1419-1212(m)	***δ***(-O-CH), ***δ***(C-C-H), ***δ***(O-H) and ***δ***(C-H)
1107 (st)	1167 (w)	1113 (m)	ν(C-O)
1035 (m)	1047 (w)	1032 (m)	ν(C-O)

ν_{s}: symmetrical stretching, ν_{as}: asymmetrical stretching, ***δ***: deformation w: weak, m: moderate, st: strong, b: broad, o: overtones.

Table S2. Band assignment of PS treatment frass spectra.

Component 1 Band Position (cm^{-1})	Component 2 Band Position (cm^{-1})	Component 3 Band Position (cm^{-1})	Type of vibration
3270 (st,b)	3267 (st,b)		ν(O-H) in Alcohol
	3150-3027 (st)	3150 (w,b)	ν(O-H) in Carboxylic Acid
	2973 (m)		ν_{as}(C-H) in Alkane
2919 (st)	2913 (st)	2922 (w)	ν_{as}(C-H) in Alkane
	2871 (m)		ν_s(C-H) in Alkane
2850 (m)	2847 (m)	2850 (w)	ν_s(C-H) in Alkane
1737 (w)	1716 (w)	1737 (w)	ν(C=O) in carbonyls
1635 (m)	1632 (st)	1611 (w)	ν(C=O) in Amide
1536 (w)	1542 (st)		ν(N-H) and ν(C-N) in Amide
	1491 (m)	1506 (w)	ν(C=C)
1458 (w)	1452 (m)	1461 (w)	***δ***(-O-CH) and ***δ***(C-C-H)
	1377-1233 (m)		***δ***(O-H) and ***δ***(C-H)
1113 (st)		1110 (st)	ν(C-O)
	1053-1029 (st)		ν(C-O)
981 (m)	951 (m)	984 (m)	ν(C-H) in Alkene
900 (st)		900 (st)	ν(C-H) in Alkene

ν_s: symmetrical stretching, ν_{as}: asymmetrical stretching, ***δ***: deformation w: weak, m: moderate, st: strong, b: broad, o: overtones.

Table S3. **Results of the sequence alignment of candidate proteins and known styrene metabolism associated proteins.**

ID Uniprot	Tipo	STYA_PSEFL (StyA)	STYB_PSEFL (StyB)	C7ACG1_RHOOP (StyA2B)	STYC_PSEFL (StyC)	StyD_PSEFL (StyD)	PHACA_EMEND (PAAH)
A0A6J1WKV6	Aldedh	15%	22%	18%	16%	42%	18%
A0A6J1WEK4	Aldedh	18%	24%	17%	17%	43%	15%
A0A6J1WXC9	Aldedh	18%	18%	18%	10%	27%	14%
A0A6J3BXC6	Aldedh	13%	10%	17%	15%	39%	15%
A0A6J3BRI2	Aldedh	15%	16%	11%	6%	41%	19%
A0A6J1WN53	Aldedh	13%	25%	15%	6%	27%	16%
A0A6J1WG03	Aldedh	14%	0%	20%	8%	30%	16%
A0A6J1WE23	FMO-like	19%	26%	21%	12%	12%	18%
A0A6J1WAJ1	FAD dep.	20%	0%	18%	17%	11%	13%
A0A6J1WQW7	FAD dep.	13%	10%	20%	0%	12%	28%
A0A6J1WVA9	Fenol oxidasa	17%	16%	15%	13%	15%	16%
A0A6J1WSG2	Fenol oxidasa	13%	18%	20%	9%	17%	18%
A0A6J1WN20	Fenol oxidasa	19%	20%	21%	9%	15%	17%
A0A6J1WH61	p450	18%	19%	16%	7%	14%	21%
A0A6J1WQ16	p450	16%	18%	15%	9%	14%	22%
A0A6J1WPN0	p450	16%	22%	18%	13%	19%	20%
A0A6J1X2I4	p450	20%	15%	16%	10%	11%	20%
A0A6J1WUC0	p450	20%	18%	13%	11%	15%	20%
A0A6J1WQ16	p450	16%	18%	17%	8%	15%	22%
A0A6J3C2W6	p450	16%	13%	14%	4%	18%	17%
A0A6J1WVU0	p450	17%	22%	19%	17%	12%	9%
A0A6J1WVU4	p450	17%	15%	19%	12%	14%	22%
A0A6J1WIH9	p450	15%	20%	16%	10%	17%	17%
A0A6J1X0S3	p450	14%	15%	17%	10%	14%	24%
A0A6J1WG08	p450	19%	14%	17%	11%	13%	23%
A0A6J1WNR2	p450	15%	11%	13%	11%	13%	16%
A0A6J1WKC9	p450	16%	10%	13%	8%	11%	21%
A0A6J1WF11	p450	18%	16%	15%	7%	13%	18%

Table S4. <u>Overexpressed proteins in the intestine of *G. mellonela* larvae fed 54 days with PS</u>.

Accession (Uniprot code)	Probable Activity (Acording to Pfam)	Target Molecule (Acording to Pfam)
A0A6J1WBL3	Hydrolase	Proteins
A0A6J3C6R1	Hydrolase	Sulfatides
A0A6J1WNC9	Translocase	diacylglycerol
A0A6J1WH61	Oxidoreductase	Unknown
A0A6J1X3R8	Hydrolase	ATP
A0A6J1X223	Transferase	Unknown
A0A6J3BV81	Oxidoreductase	Monosaccharide
A0A6J3BWU6	Transferase	Lipids
A0A6J1X679	Hydrolase	DNA
A0A6J3BZ33	Transferase	
A0A6J1WL78	Hydrolase	Peptides
A0A6J1X165	Transferase	lipopolysaccharide
A0A6J1WHN6	Hydrolase	DNA
A0A6J1WES0	Oxidoreductase	Porphyrins
A0A6J1WPR0	Transferase	Phosphate
A0A6J3BUE1	Transferase	Phosphate
A0A6J1WP53	Hydrolase	
A0A6J3C0A8	Ligase	DNA
A0A6J3BRP8	Transferase	Phosphate
A0A6J3CFW6	Translocase	Choline
A0A6J3C684	Hydrolase	GTP
A0A6J3BSY6	Hydrolase	DNA
A0A6J1WTS8	Hydrolase	GTP
A0A6J1WTF0	Hydrolase	GTP
A0A6J1WQ16	Oxidoreductase	Unknown
A0A6J1X0N3	Hydrolase	GTP
A0A6J1WTF9	Hydrolase	
A0A6J1X0E1	Hydrolase	GTP
A0A6J1WT63	Hydrolase	
A0A6J1WNV0	Oxidoreductase	Lipids
A0A6J3C1Q1	Translocase	Monocarboxylate
A0A6J1WS19	Hydrolase	Unknown
A0A6J1WTT5	Hydrolase	
A0A6J1WQM2	Translocase	Unknown
A0A6J3CAP1	Hydrolase	
A0A6J1W800	Ligase	Asparagine
A0A6J3CCQ5	Hydrolase	
A0A6J1WXN8	Translocase	Trealose
A0A6J1X3U8	Transferase	Ubiquitin
A0A6J1WXP3	Translocase	Trealose
A0A6J1WT26	Transferase	Aminoacids
A0A6J1W8I8	Translocase	monocarboxylate

A0A6J1WL17	Hydrolase	Lipids
A0A6J1X4N6	Translocase	Trealose
A0A6J3C7Q5	Unknown	isoprenoids
A0A6J3C122	Translocase	HCO3-
A0A6J1WSQ9	Oxidoreductase	Lipids
A0A6J1WZ81	Transferase	Methyl-group
A0A6J1WX37	Translocase	Trealose
A0A6J3CBT9	Translocase	Trealose
A0A6J1X2B6	Transferase	Oligosaccharide
A0A6J1X0A7	Translocase	monocarboxylate
A0A6J3CCP9	Hydrolase	Histone
A0A6J1WYT9	Transferase	
A0A6J3BTT6	Hydrolase	
A0A6J1WJR3	Transferase	
A0A6J1WV21	Hydrolase	GTP
A0A6J1X314	Isomerase	Glucose
A0A6J1WQK4	Ligase	
A0A6J3C4Z1	Transferase	
A0A6J1WX91	Oxidoreductase	
A0A6J1WGI4	Transferase	RNA
A0A6J1WBH6	Transferase	Phosphoenolpyruvate
A0A6J1X2I4	Oxidoreductase	Unknown
A0A6J3CGY3	Transferase	UDP-sugar
A0A6J1X7U1	Translocase	Unknown
A0A6J1WF71	Hydrolase	GTP
A0A6J1WE23	Oxidoreductase	Unknown
A0A6J1WUU1	Translocase	Unknown
A0A6J1WRR7	Oxidoreductase	Hydrocarbons
A0A6J1X4P2	Hydrolase	DNA
A0A6J1W6F7	Hydrolase	DNA
A0A6J1WLY1	Transferase	Manose
